# The effects of an 8-week French Contrast Training program on lower limb strength and power in elite martial arts athletes

**DOI:** 10.3389/fspor.2025.1686891

**Published:** 2025-10-24

**Authors:** Hao Chen, Ziren Zhao, Xin Zheng, Liquan Cao, Jingtao Du, Zhongtao Yu

**Affiliations:** ^1^ Department of Sports Health and Rehabilitation, Tianjin Vocational College of Sports, Tianjin, China; ^2^ College of Physical Education and Health Science, Chongqing Normal University, Chongqing, China; ^3^ Department of Sports Health and Rehabilitation, Tianjin University of Sport, Tianjin, China; ^4^ College of Sports and Health, Chengdu University of Traditional Chinese Medicine, Chengdu, Sichuan, China; ^5^ Tianjin Institute of Sports Science, Tianjin Sports Comprehensive Guarantee Center, Tianjin, China

**Keywords:** French Contrast Training, elite martial arts athletes, isometric mid-thigh pull, countermovement jump, squat jump, elasticity index, dynamic strength index

## Abstract

**Background:**

Lower limb strength and power are critical for martial arts athletes to perform complex movements such as aerial outward swings. French Contrast Training (FCT), which integrates heavy compound exercises, plyometrics, and assisted plyometric movements into a single session, has been proposed to elicit superior neuromuscular adaptations. However, the effectiveness of FCT in elite martial arts athletes remains unclear.

**Objective:**

This study aimed to investigate the effects of an 8-week FCT program on lower limb strength and power in elite martial arts athletes.

**Methods:**

In total, 24 elite male martial arts athletes were randomly assigned to the FCT group (*n* = 12) or the control group (*n* = 12). Both groups completed an 8-week (twice a week) training program. The FCT protocol included the following four sequential exercises per session: 85% one-repetition maximum (1RM) back squats, countermovement jumps (CMJs), 30% 1RM squat jumps (SJs), and band-assisted jumps. The control group performed traditional resistance training for the same muscle groups. The pre- and post-intervention assessments included isometric mid-thigh pulls [IMTPs; maximal force output (MFO), relative MFO, and rate of force development (RFD)], CMJs, SJs with jump height, peak power output (PPO), and mean power output (MPO), elasticity index (EI), and dynamic strength index (DSI). All the data were analyzed using the linear mixed model. Effect sizes were calculated using Cohen's *d*. Statistical significance was set at *p* < 0.05.

**Results:**

The FCT group showed significantly greater improvements than the control group in IMTP (MFO: *p* < 0.001, *d* = 1.66; relative MFO: *p* = 0.001, *d* = 1.51; RFD: *p* = 0.001, *d* = 1.52), CMJ (jump height: *p* = 0.011, *d* = 1.14; PPO: *p* < 0.001, *d* = 1.61; MPO: *p* = 0.013, *d* = 1.11), SJ (jump height: *p* = 0.019, *d* = 1.03; PPO: *p* = 0.043, *d* = 0.88; MPO: *p* < 0.001, *d* = 1.63), EI (*p* = 0.521, *d* = −0.27), and DSI (*p* < 0.001, *d* = 2.07). No adverse events were reported.

**Conclusions:**

This study provides preliminary evidence that French Contrast Training effectively enhances lower body strength and power in elite martial arts athletes.

## 1 Introduction

Lower limb strength and power are critical for martial arts athletes to succeed in complex movements (e.g., aerial outward swing) (1). Lower limb strength and power govern movement amplitude, execution speed, and postural control (2). Specifically, maximal strength provides take-off impulse, whereas rapid force development and eccentric braking reduce ground contact time, enable rapid acceleration, and stabilize landings in acrobatic skills (3–7). Consequently, lower limb strength and power underpin technical elements such as the aerial outward swing and align with competition criteria. Traditional training methods (e.g., resistance training and ballistic training) are integral to martial arts athletes' training programs, which elicit morphological and functional muscular adaptations and positively influence strength and jumping performance (8). Khazaei et al. reported that 8 weeks of traditional resistance training significantly improved the maximal strength, vertical jump, and sprint speed of elite taekwondo athletes (9). Lecce et al. separately reported that 4 weeks of resistance training and ballistic training (maximal intent) significantly improved elite athletes' propulsive power and propulsive velocity at the maximal and mean levels, rate of force development (RFD), and impulse (10, 11). However, resistance training predominantly targets the high-force, low-velocity region of the force–velocity curve (F–V curve). It provides limited stimulus to the high-velocity region, which constitutes a methodological limitation for developing rapid production and stretch-shortening cycle (SSC) efficiency (12). French Contrast Training (FCT) has garnered considerable attention for its potential to enhance maximal strength and power by strategically combining high-load, low-to moderate-load, and plyometric exercises in a single session, thereby stimulating adaptations across the full F–V curve (13–18). For example, a 6-week FCT program for professional soccer players has been shown to effectively improve their vertical jump, 30 m sprint, and dynamic balance compared to routine tactical drills (15). Similarly, Elbadry et al. demonstrated that an 8-week FCT program significantly enhanced the countermovement jump (CMJ), medicine ball throw, and triple jump performance of female college athletes. In addition, Rebelo et al. reported marked improvements in maximal strength, reactive strength index, and CMJ in recreational young athletes following FCT (19). These findings support the efficacy of FCT as an advanced training strategy for enhancing lower limb power among various athletic populations compared to routine training. However, the effects of FCT on athletes' power in comparison to traditional resistance training remain unclear.

FCT integrates the following four exercise phases into a single session: (1) a heavy compound exercise [e.g., 80%–90% one-repetition maximum (1RM) back squat], (2) a plyometric exercise (e.g., vertical jump), (3) a light-to-moderate load exercise (e.g., ∼30% 1RM barbell weighted jumps), and (4) an assisted plyometric exercise (e.g., band-assisted jump) (20, 21). This structured protocol systematically stimulates adaptations across the F–V curve to optimize neuromuscular function (22, 23). In addition, based on post-activation performance enhancement (PAPE) principles, this method integrates contrasting loads and movement velocities to stimulate the central nervous system and optimize force output (24). While FCT has demonstrated promising results in various athletic populations, limited research exists in martial arts athletes, whose performance relies heavily on lower limb strength and power for kicks, takedowns, and rapid directional changes.

This study aims to address this gap by evaluating the effects of an 8-week FCT protocol on lower limb strength and power in martial arts athletes. To evaluate these effects, we assessed force output, RFD, vertical jump height, vertical jump power output, elasticity index (EI), and dynamic strength index (DSI), thereby providing empirical evidence for FCT as a targeted conditioning strategy in martial arts. We hypothesized that FCT would elicit significantly greater improvements in maximal force output (MFO), relative MFO, RFD, jump height, peak power output (PPO), mean power output (MPO), EI, and DSI.

## 2 Materials and methods

### 2.1 Participants

Based on pre-experimental data (mean effect size for CMJ and squat jump (SJ) height: Cohen's *d* = 0.442; correlation coefficient *r* = 0.984), we calculated, using G*Power software (ANOVA analysis, *f* = 0.41, *α* = 0.05, power = 0.95, correlation coefficient *r* = 0.85) (Supplementary Table S1), that this study required 10 participants. In total, 24 elite martial arts athletes participated in this study. The participants were randomly assigned to either the FCT group (*n* = 12) or the control (CON) group (*n* = 12) (Table 1). All the participants were healthy, non-smokers, and not taking any medications or supplements. The eligibility criteria were as follows: male athletes who had competed in the quarterfinals of the National Junior Competition or achieved a top-three ranking in individual events or a top-two ranking in team events at the provincial level or higher. The study was approved by the ethics committee of Tianjin University of Sport (TJUS 2025-039), and all the procedures were conducted according to the Declaration of Helsinki. Before participating, all the athletes were fully informed of the potential benefits and risks of the study and provided written informed consent.

**Table 1:** Baseline characteristics of the participants by group.

Characteristic	FCT (*n* = 12)	CON (*n* = 12)	*t*	*p*
Height (cm)	162.3 ± 6.1	163.4 ± 4.6	0.831	0.415
Body mass (kg)	63.8 ± 8.3	60.8 ± 5.4	1.052	0.304
Training age (years)	10.5 ± 3.2	8.5 ± 2.8	1.633	0.117
1RM squat (kg)	158.8 ± 22.0	176.7 ± 15.4	−2.313	0.030
MFO (N)	2,197.86 ± 628.34	2,191.24 ± 580.56	0.027	0.979
RMFO (N/kg)	3.65 ± 0.612	3.61 ± 0.53	0.177	0.861
RFD (N/s)	3,264.73 ± 812.10	3,243.99 ± 735.48	0.066	0.948
CMJ height (cm)	48.28 ± 8.49	46.48 ± 11.52	0.436	0.667
CMJ PPO (W)	4,070.67 ± 1,178.53	4,069.17 ± 1,379.79	0.003	0.998
CMJ MPO (W)	2,209.75 ± 604.38	2,208.58 ± 641.03	0.005	0.996
SJ height (cm)	47.84 ± 8.73	47.68 ± 11.46	0.038	0.970
SJ PPO (W)	4,816.50 ± 972.68	4,815.58 ± 1,161.07	0.002	0.998
SJ MPO (W)	2,297.83 ± 641.95	2,297.75 ± 766.65	0.001	0.998
EI	3.09 ± 23.58	−0.14 ± 24.53	0.329	0.746
DSI	0.96 ± 0.09	0.94 ± 0.09	0.452	0.656

CMJ, countermovement jump; DSI, dynamic strength index; EI, elasticity index; IMTP, isometric mid-thigh pull; MFO, maximal force output; MPO, mean power output; PPO, peak power output; RMFO, relative maximal force output; SJ, squat jump; 1RM, one-repetition maximum.

Values are mean ± SD.

### 2.2 Study design

**Figure 1:**
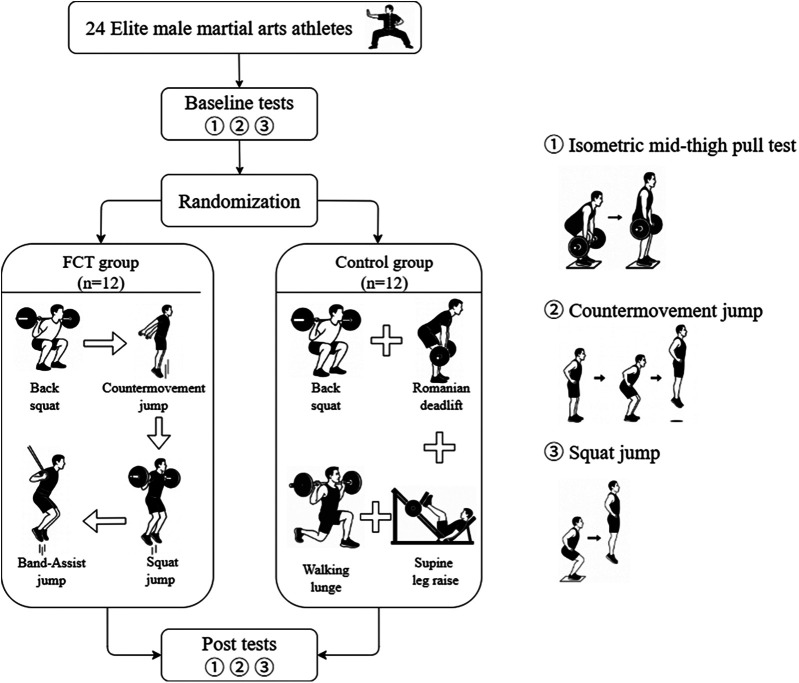
Flow chart of the experiment.

This study was an 8-week, single-blind (assessor-blind) randomized controlled trial (Figure 1). The participants were randomly assigned to either the FCT group or the control group using a computer-generated randomization sequence. Both groups completed their training protocols twice weekly, with at least 48 h of rest between sessions. The intervention was conducted during the preparatory phase of the competitive season. All the participants underwent a familiarization session before the intervention to learn the training procedures and testing protocols. Certified strength and conditioning coaches delivered standardized instruction to ensure consistent training implementation and evaluation. To control for potential circadian rhythm effects, all testing sessions were conducted at the same time of day for each participant. We used a digital thermohydrometer (SW-572, SNDWAY, China) to measure the environmental conditions, with an average temperature of 21.2 ± 0.3 °C and an average humidity of 29.0% ± 0.4%. The participants were instructed to refrain from strenuous physical activity for 48 h before each testing session and to avoid alcohol and caffeine intake within 24 h before testing.

#### 2.2.1 Training protocol

**Figure 2:**
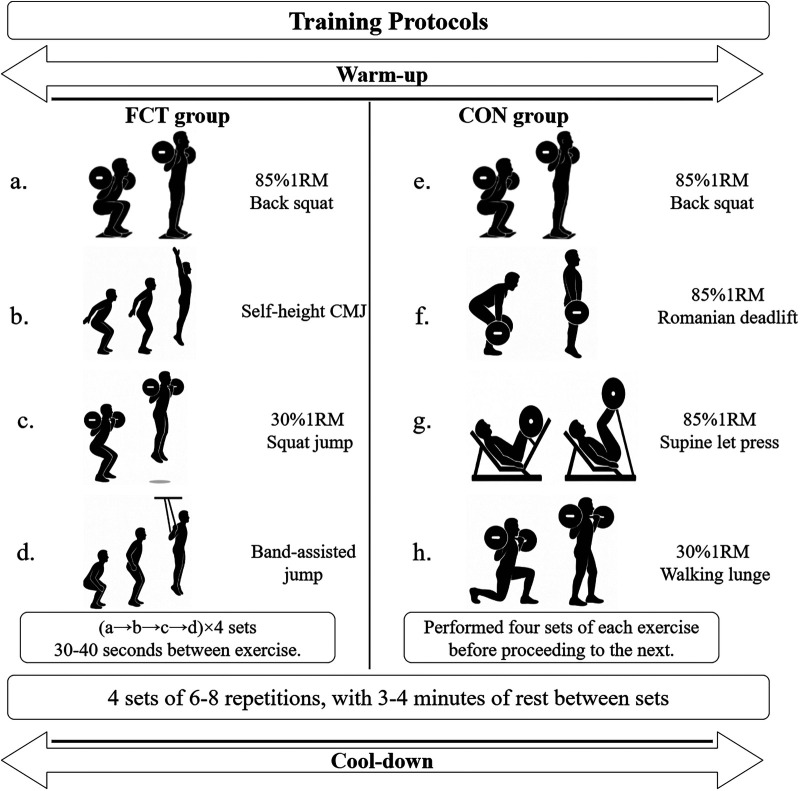
Schematic of the experimental protocol. **(a, b, c, d)** FCT training protocols; **(e, f, g, h)** CON training protocols.

The participants in the FCT group performed a structured four-phase training protocol, targeting distinct regions along the F–V curve. This protocol consisted of (1) a heavy compound exercise (85% 1RM back squat), (2) a plyometric exercise (CMJ with arm swing), (3) a light-to-moderate load exercise (30% 1RM SJ)], and (4) an assisted plyometric exercise (band-assisted jump). The participants in the control group engaged in traditional resistance training matched to the FCT protocol for total training duration and targeted muscle groups, including (1) 85% 1RM back squats, (2) 85% 1RM Romanian deadlifts, (3) 85% 1RM supine leg raises, and (4) 30% 1RM walking lunges. Both groups completed four sets of six to eight repetitions per exercise, with 3–4 min of rest between sets. All the participants began each training session with a standardized 10-min warm-up (Supplementary Table S2) and finished with a 5-min cooldown (Figure 2). Training loads were calculated from baseline 1RM values and were not adjusted during the 8-week intervention. Apart from the intervention, all the participants completed sport-specific technical training, with equivalent content and duration.

Adherence was defined as sessions attended divided by sessions planned, multiplied by 100%, at both the participant and group levels.

#### 2.2.2 Outcomes measurement

##### 2.2.2.1 Isometric mid-thigh pull test

We utilized the isometric mid-thigh pull test (IMTP) to measure the force output and RFD of the participants. The IMTP test procedure (25) was as follows: (1) the assessor instructed the participants to stand on a force plate (Kistler Group, ≥1,000 Hz, Switzerland) with their feet shoulder-width apart and to fix the bar at mid-thigh height to imitate the second pull position of a high clean exercise; (2) the knee and hip angles of the participants were standardized at approximately 130° and 145° and verified using a handheld goniometer; and (3) the assessor instructed the participants to gradually build tension and then pull maximally “as fast and hard as possible” for 3–5 s while keeping their body rigid and maintaining complete foot contact. Verbal encouragement was provided throughout the test to ensure maximal effort. Each participant performed three maximal attempts with a 2-min rest between trials. The best results for MFO (N), relative MFO (N/kg), and peak RFD (N/s) were recorded for the analysis.

##### 2.2.2.2 Countermovement jump test

We utilized the CMJ to evaluate the participants' lower limb power under dynamic conditions. The CMJ test procedure (26) was as follows: (1) the participants stood upright on a force platform (Kistler Group, ≥1,000 Hz, Switzerland) with their hands placed firmly on their iliac crests to minimize arm swing; and (2) upon receiving an auditory signal, the participants rapidly descended into a squat position and immediately performed a vertical jump. Each participant performed three maximal attempts with a 1-min rest between trials. Jump height was derived using the impulse–momentum method; the center-of-mass velocity was obtained by integrating the net vertical force (*Fz − BW*)/*m* [initial condition v (0) = 0], and height was computed from take-off velocity using the following equation: *h* *=* *v2TO/*(*2 g*) (5). The best results for jump height (cm), PPO (W), and MPO (W) were recorded for the analysis.

##### 2.2.2.3 Squat jump test

We utilized the SJ test to evaluate participants' lower limb concentric power without the influence of the stretch-shortening cycle. The SJ test procedure (26) was as follows: (1) the participants started from a static squat position on a force platform (Kistler Group, ≥1,000 Hz, Switzerland) with their knees flexed at 90°, which was verified using a handheld goniometer; and (2) the participants executed a maximal vertical jump while keeping their hands placed on their iliac crests to minimize arm swing. Each participant performed three maximal attempts with a 1-min rest between trials. Jump height was calculated using the impulse–momentum method with the same integration procedure as CMJ. The best results for jump height (cm), PPO (W), and MPO (W) were recorded for the analysis.

##### 2.2.2.4 EI

We calculated the EI to assess participants' ability to utilize the SSC during jumping. EI was determined using the following formula: (CMJ − SJ)/SJ × 100% (27). A higher EI signifies greater efficiency in SSC utilization.

##### 2.2.2.5 DSI

We calculated the DSI to assess the balance between the participants’ maximal isometric force capacity and ballistic force expression. The DSI was determined using the following formula: CMJ peak force/IMTP MFO (28). A higher DSI reflects a greater translation of maximal isometric strength into dynamic explosive performance.

### 2.3 Statistical analysis

We present all the data as mean ± standard deviation (SD) and checked for normality using the Shapiro–Wilk test. We used the independent *t*-test to compare baseline variables between the FCT and CON groups (Table 1). We applied the linear mixed model (LMM) with fixed effects for group, time, and their interaction (group × time), and a random intercept for participants (REML, Satterthwaite, UK). We focused on the group × time interaction and reported the difference in change (ΔFCT − ΔCON) with 95% confidence intervals (CIs). When relevant, we examined simple effects with Sidak adjustment. We used the independent *t*-test to compare the percentage change (Δ%) between the groups and reported the results with 95% CIs. We calculated Cohen's d to ascertain the effect size, which was categorized as trivial (<0.20), small (0.20–0.49), moderate (0.50–0.79), or large (≥0.80). A *p-*value < 0.05 was considered statistically significant. All the analyses were performed using SPSS (version 27.0, IBM Statistics, Chicago, IL, USA).

## 3 Results

All the participants completed the 8-week training as prescribed in both groups, resulting in 100% session completion.

### 3.1 IMTP

#### 3.1.1 MFO

**Figure 3:**
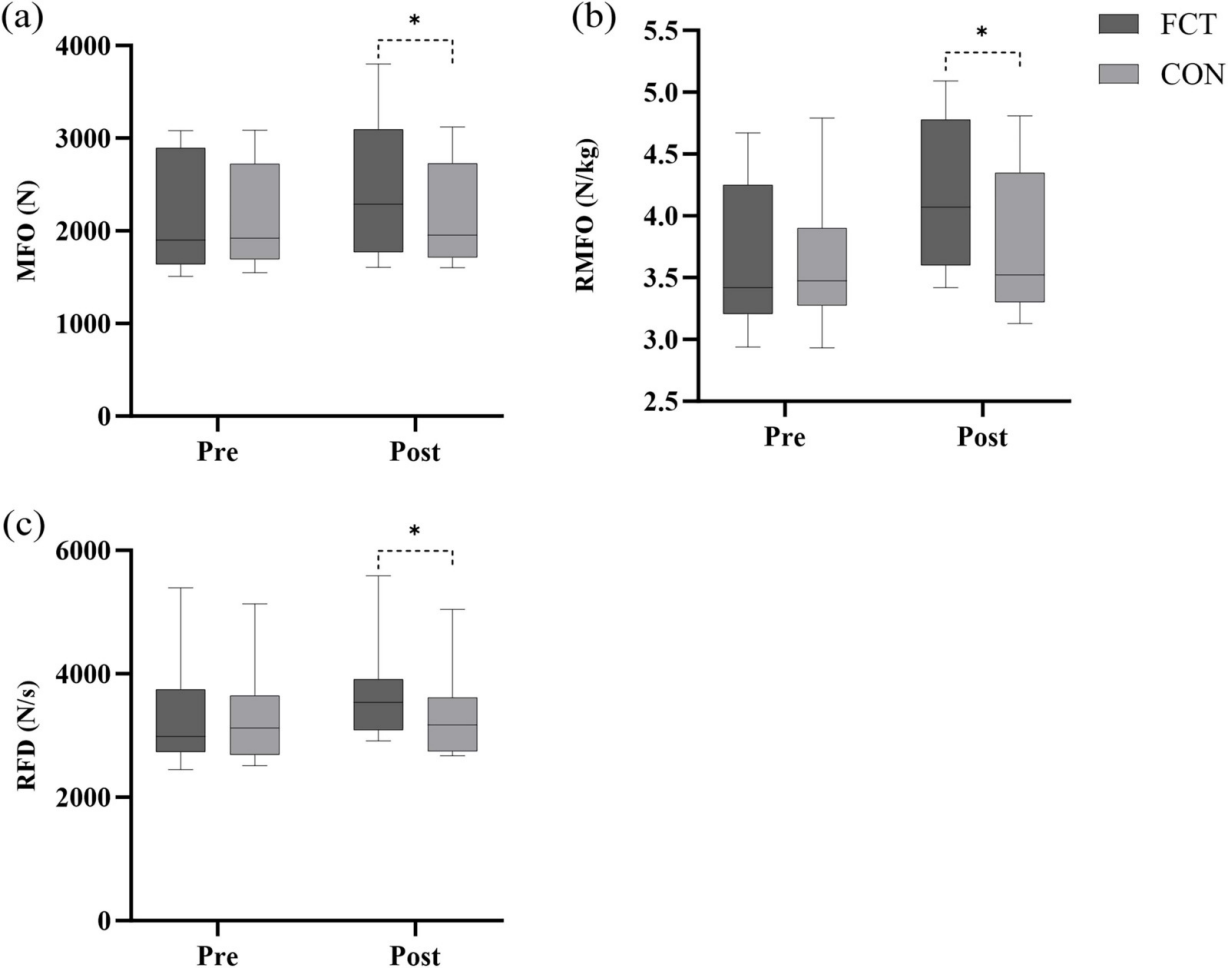
Effect of FCT on the IMTP. **(a)** IMTP MFO, **(b)** IMTP RMFO, **(c)** IMTP RFD.

A significant interaction effect between time and group was observed (F = 12.63, *p* = 0.002). The adjusted difference in change was 237.63 (*p* = 0.002, 95% CI: 98.97–376.29). The main effect of time showed a significant difference (F = 16.37, *p* < 0.001). The main effect of the group showed a significant difference (F = 13.00, *p* = 0.002) (Figure 3a; Table 2).

**Table 2:** The effects of the FCT and CON interventions on the athletic performance of the participants.

Variable	Group	Pre-intervention	Post-intervention	Δ% (mean)	*p*		
					Time	Group	Interaction
IMTP	MFO (N)						
	FCT	2,197.86 ± 628.34	2,451.92 ± 754.61	11.16	<0.001	0.002	0.002
	CON	2,191.24 ± 580.56	2,207.67 ± 570.64	0.96			
	RMFO (N/kg)						
	FCT	3.65 ± 0.612	4.15 ± 0.60	14.17	<0.001	<0.001	<0.001
	CON	3.61 ± 0.53	3.74 ± 0.57	3.66			
	RFD (N/s)						
	FCT	3,264.73 ± 812.10	3,645.02 ± 717.92	13.08	<0.001	<0.001	<0.001
	CON	3,243.99 ± 735.48	3,261.19 ± 675.69	0.93			
CMJ	Height (cm)						
	FCT	48.28 ± 8.49	50.41 ± 8.51	4.57	<0.001	0.002	0.002
	CON	46.48 ± 11.52	47.08 ± 11.29	1.47			
	PPO (W)						
	FCT	4,070.67 ± 1,178.53	4,142.42 ± 1,164.64	2.00	<0.001	<0.001	<0.001
	CON	4,069.17 ± 1,379.79	4,076.75 ± 1,371.93	0.28			
	MPO (W)						
	FCT	2,209.75 ± 604.38	2,266.92 ± 617.47	2.64	0.002	0.010	0.008
	CON	2,208.58 ± 641.03	2,214.67 ± 621.76	0.58			
SJ	Height (cm)						
	FCT	47.84 ± 8.73	50.02 ± 7.98	4.95	<0.001	0.003	0.007
	CON	47.68 ± 11.46	48.25 ± 11.12	1.50			
	PPO (W)						
	FCT	4,816.50 ± 972.68	5,067.08 ± 924.3	6.04	<0.001	0.014	0.014
	CON	4,815.58 ± 1,161.07	4,832.08 ± 1,149.29	0.22			
	MPO (W)						
	FCT	2,297.83 ± 641.95	2,461.92 ± 632.08	7.89	<0.001	<0.001	<0.001
	CON	2,297.75 ± 766.65	2,302.5 ± 761.99	0.36			
EI	FCT	3.09 ± 23.58	2.37 ± 21.33	−0.72	0.858	0.542	0.521
	CON	−0.14 ± 24.53	0.27 ± 26.81	0.41			
DSI	FCT	0.96 ± 0.09	1.13 ± 0.10	17.83	<0.001	<0.001	<0.001
	CON	0.94 ± 0.09	0.96 ± 0.08	2.23			

CMJ, countermovement jump; DSI, dynamic strength index; EI, elasticity index; IMTP, isometric mid-thigh pull; MFO, maximal force output; MPO, mean power output; PPO, peak power output; RMFO, relative maximal force output; SJ, squat jump.

**Figure 4:**
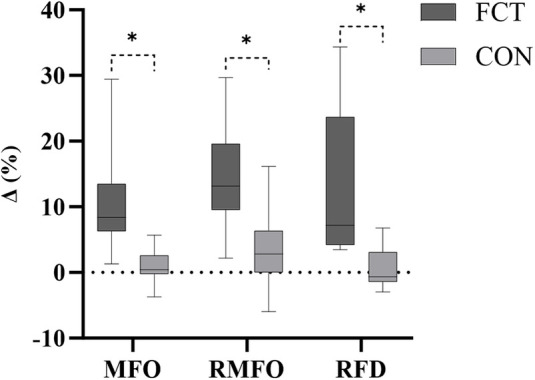
The IMTP growth rate.

An independent *t*-test comparing the Δ% in MFO revealed a significant difference between the groups (*t* = 4.07, *p* < 0.001, 95% CI: 5.01–15.40, *d* = 1.66, large effect) (Figure 4).

#### 3.1.2 Relative MFO

A significant interaction effect between time and group was observed (F = 15.38, *p* < 0.001). The adjusted difference in change was 0.369 (*p* < 0.001, 95% CI: 0.174–0.564). The main effect of time showed a significant difference (F = 44.19, *p* < 0.001). The main effect of the group showed a significant difference (F = 15.14, *p* < 0.001) (Figure 3b; Table 2).

An independent *t*-test comparing the Δ% in relative MFO revealed a significant difference between the groups (*t* = 3.70, *p* = 0.001, 95% CI: 4.61–16.40, *d* = 1.51, large effect) (Figure 4).

#### 3.1.3 RFD

A significant interaction effect between time and group was observed (F = 18.90, *p* < 0.001). The adjusted difference in change was 363.10 (*p* < 0.001, 95% CI: 189.88–536.32). The main effect of time showed a significant difference (F = 22.65, *p* < 0.001). The main effect of the group showed a significant difference (F = 24.16, *p* < 0.001) (Figure 3c; Table 2).

An independent *t*-test comparing the Δ% in RFD revealed a significant difference between the groups (*t* = 3.72, *p* = 0.001, 95% CI: 5.39–18.92, *d* = 1.52, large effect) (Figure 4).

### 3.2 CMJ

#### 3.2.1 Height

**Figure 5:**
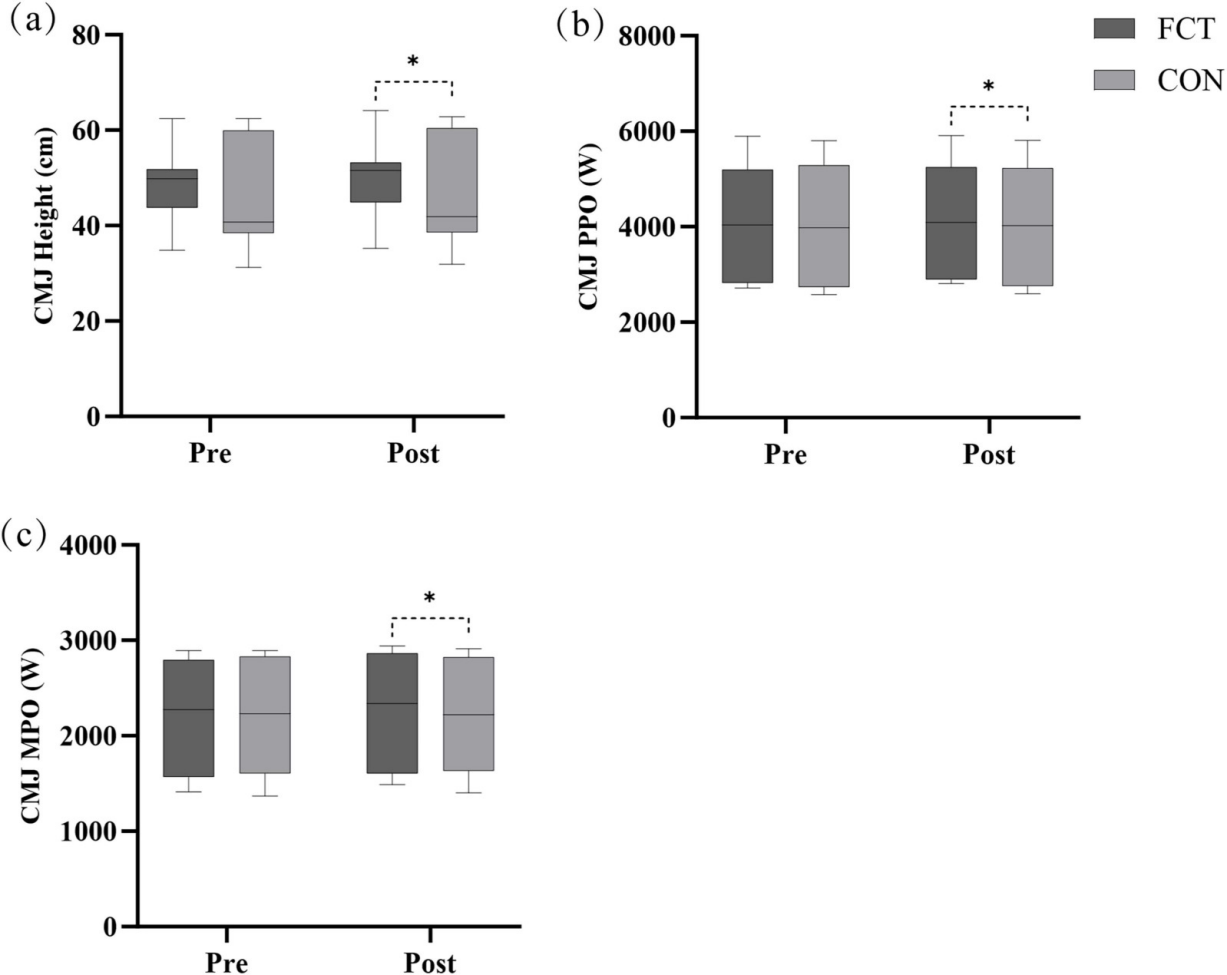
Effect of FCT on the CMJ. **(a)** CMJ Height, **(b)** CMJ PPO, **(c)** CMJ MPO.

A significant interaction effect between time and group was observed (F = 11.07, *p* = 0.002). The adjusted difference in change was 1.53 (*p* = 0.002, 95% CI: 0.604–2.463). The main effect of time showed a significant difference (F = 35.19, *p* < 0.001). The main effect of the group showed a significant difference (F = 11.22, *p* = 0.002) (Figure 5a; Table 2).

**Figure 6:**
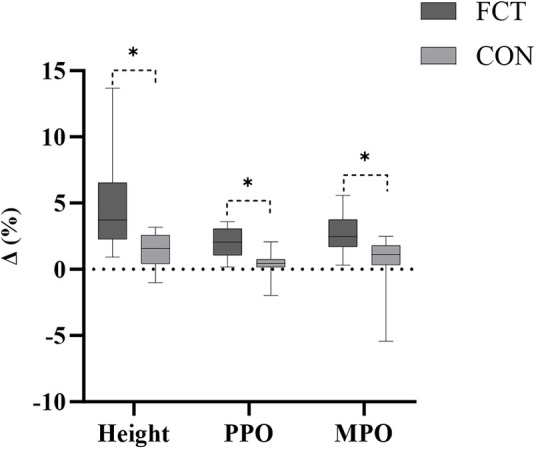
The CMJ growth rate.

An independent *t*-test comparing the Δ% in CMJ height revealed a significant difference between the groups (*t* = 2.80, *p* = 0.011, 95% CI: 0.80–5.40, *d* = 1.14, large effect) (Figure 6).

#### 3.2.2 PPO

A significant interaction effect between time and group was observed (F = 17.55, *p* < 0.001). The adjusted difference in change was 64.17 (*p* < 0.001, 95% CI: 32.40–95.94). The main effect of time showed a significant difference (F = 26.82, *p* < 0.001). The main effect of the group showed a significant difference (F = 13.87, *p* < 0.001) (Figure 5b; Table 2).

An independent *t*-test comparing the Δ% in CMJ PPO revealed a significant difference between the groups (*t* = 3.94, *p* < 0.001, 95% CI: 0.82–2.62, *d* = 1.61, large effect) (Figure 6).

#### 3.2.3 MPO

A significant interaction effect between time and group was observed (F = 8.39, *p* = 0.008). The adjusted difference in change was 51.08 (*p* = 0.008, 95% CI: 14.50–87.66). The main effect of time showed a significant difference (F = 12.86, *p* = 0.002). The main effect of the group showed a significant difference (F = 8.11, *p* = 0.010) (Figure 5c; Table 2).

An independent *t*-test comparing the Δ% in CMJ MPO revealed a significant difference between the groups (*t* = 2.71, *p* = 0.013, 95% CI: 0.48–3.63, *d* = 1.11, large effect) (Figure 6).

### 3.3 SJ

#### 3.3.1 Height

**Figure 7:**
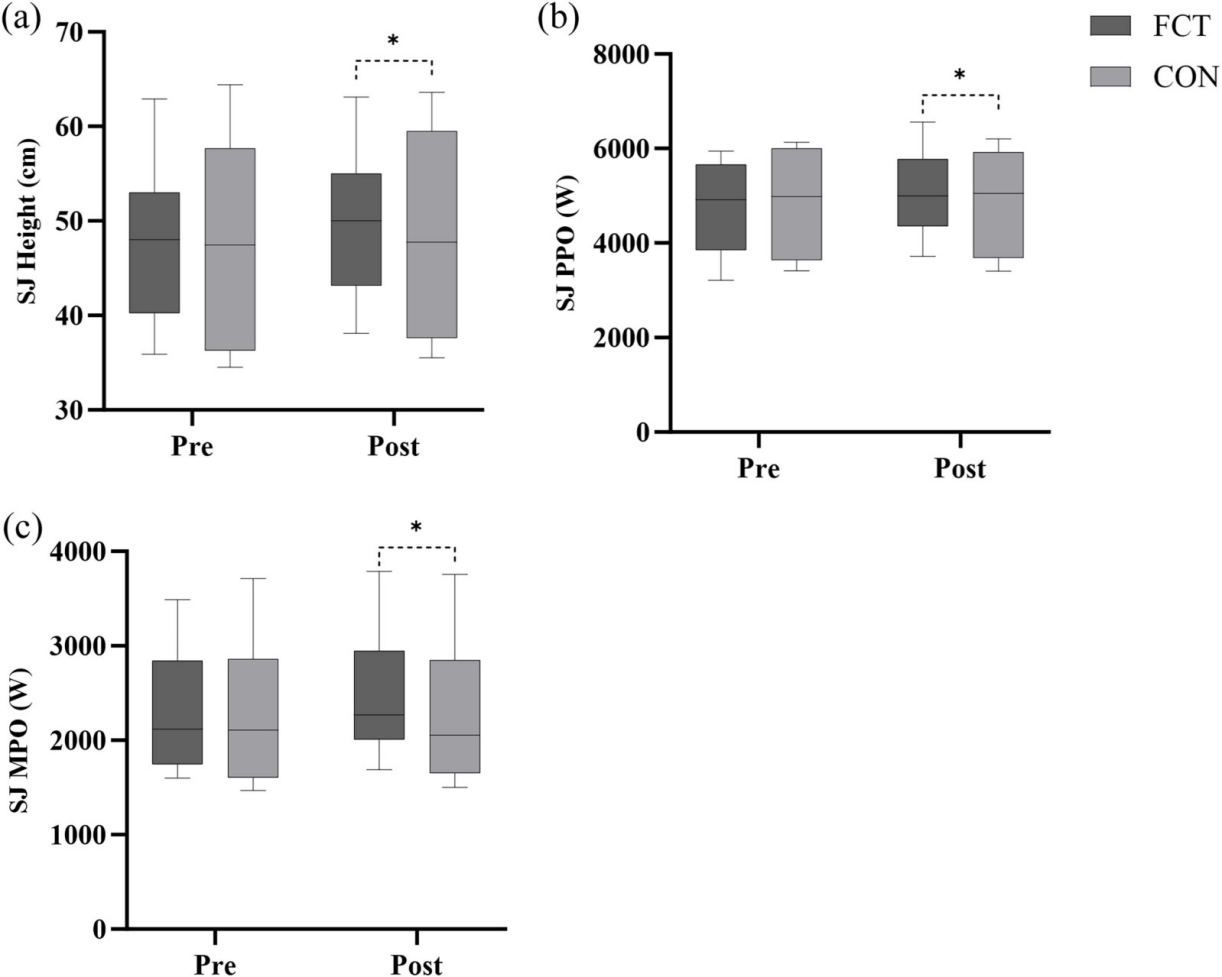
Effect of FCT on the SJ. **(a)** SJ Height, **(b)** SJ PPO, **(c)** SJ MPO.

A significant interaction effect between time and group was observed (F = 8.14, *p* = 0.007). The adjusted difference in change was 1.61 (*p* = 0.007, 95% CI: 0.47–2.75). The main effect of time showed a significant difference (F = 23.95, *p* < 0.001). The main effect of the group showed a significant difference (F = 9.62, *p* = 0.003) (Figure 7a; Table 2).

**Figure 8:**
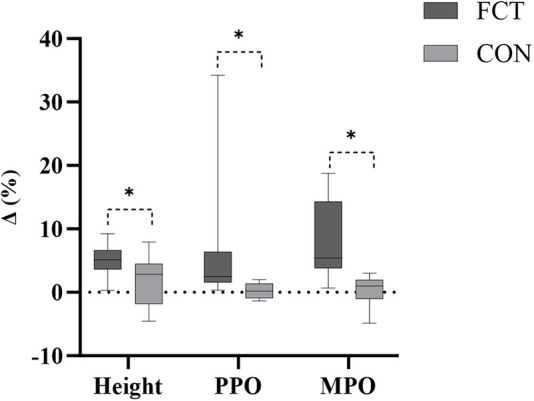
The SJ growth rate.

An independent *t*-test comparing the Δ% in SJ height revealed a significant difference between the groups (*t* = 2.52, *p* = 0.019, 95% CI: 0.61–6.29, *d* = 1.03, large effect) (Figure 8).

#### 3.3.2 PPO

A significant interaction effect between time and group was observed (F = 7.02, *p* = 0.014). The adjusted difference in change was 243.08 (*p* = 0.014, 95% CI: 53.36–432.81). The main effect of time showed a significant difference (F = 7.96, *p* < 0.010). The main effect of the group showed a significant difference (F = 7.11, *p* = 0.014) (Figure 7b; Table 2).

An independent *t*-test comparing the Δ% in SJ PPO revealed a significant difference between the groups (*t* = 2.15, *p* = 0.043, 95% CI: 0.20–11.44, *d* = 0.88, large effect) (Figure 8).

#### 3.3.3 MPO

A significant interaction effect between time and group was observed (F = 21.95, *p* < 0.001). The adjusted difference in change was 159.33 (*p* < 0.001, 95% CI: 88.80–229.87). The main effect of time showed a significant difference (F = 24.64, *p* < 0.001). The main effect of the group showed a significant difference (F = 21.38, *p* < 0.001) (Figure 7c; Table 2).

An independent *t*-test comparing the Δ% in SJ MPO revealed a significant difference between the groups (*t* = 3.98, *p* < 0.001, 95% CI: 3.61–11.45, *d* = 1.63, large effect) (Figure 8).

### 3.4 EI

**Figure 9:**
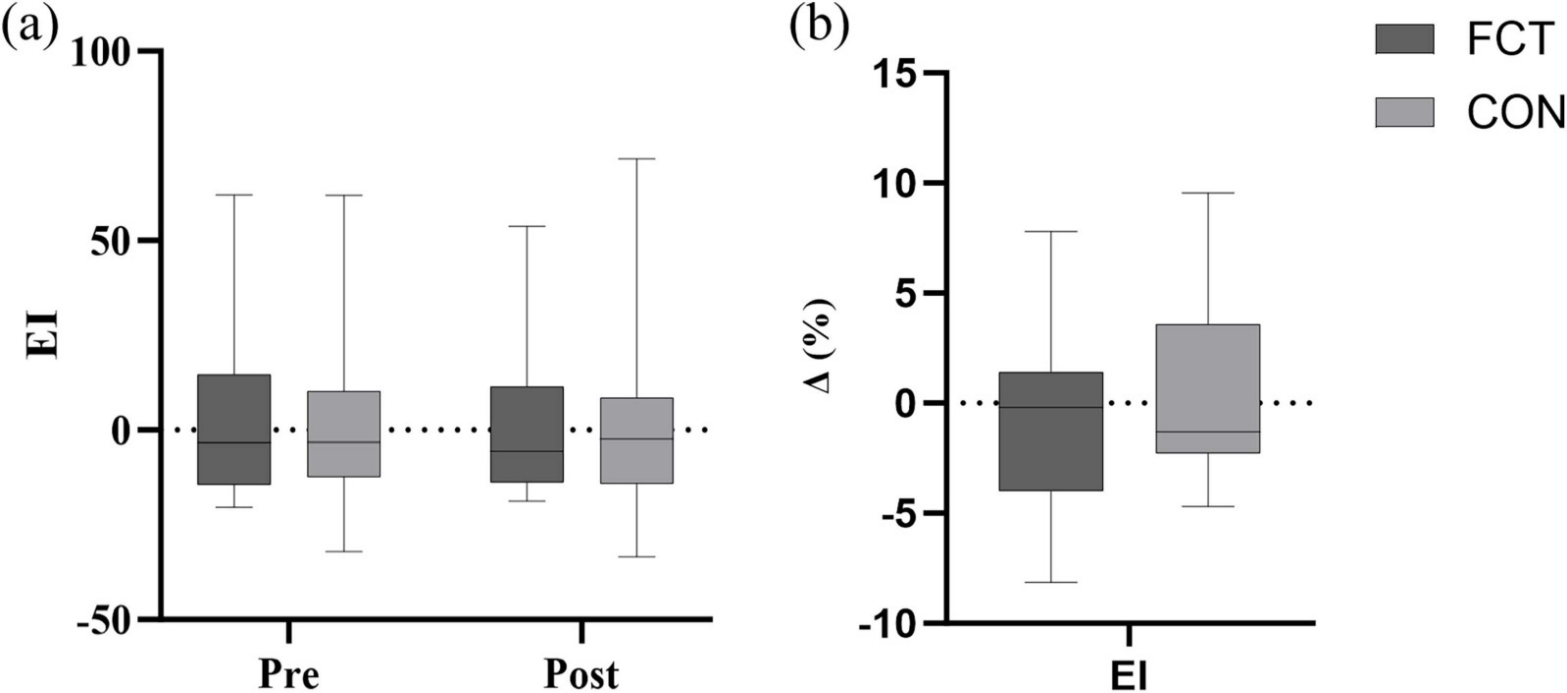
Effect of FCT on the EI and the EI growth rate. **(a)** EI, **(b)** growth rate.

A non-significant interaction effect between time and group was observed (F = 0.42, *p* = 0.521). The adjusted difference in change was 1.12 (*p* = 0.521, 95% CI: −2.45–4.70). The main effect of time showed a non-significant difference (F = 0.03, *p* = 0.858). The main effect of the group showed a non-significant difference (F = 0.39, *p* = 0.542) (Figure 9a). Due to normality violations, a Mann–Whitney *U* test was used to validate these findings (U = 72.00, *p* = 0.99) (Table 2).

An independent *t*-test comparing the Δ% in EI revealed a non-significant difference between the groups (*t* = −0.65, *p* = 0.521, 95% CI: −4.70–2.45, *d* = −0.27, small effect) (Figure 9b).

### 3.5 DSI

**Figure 10:**
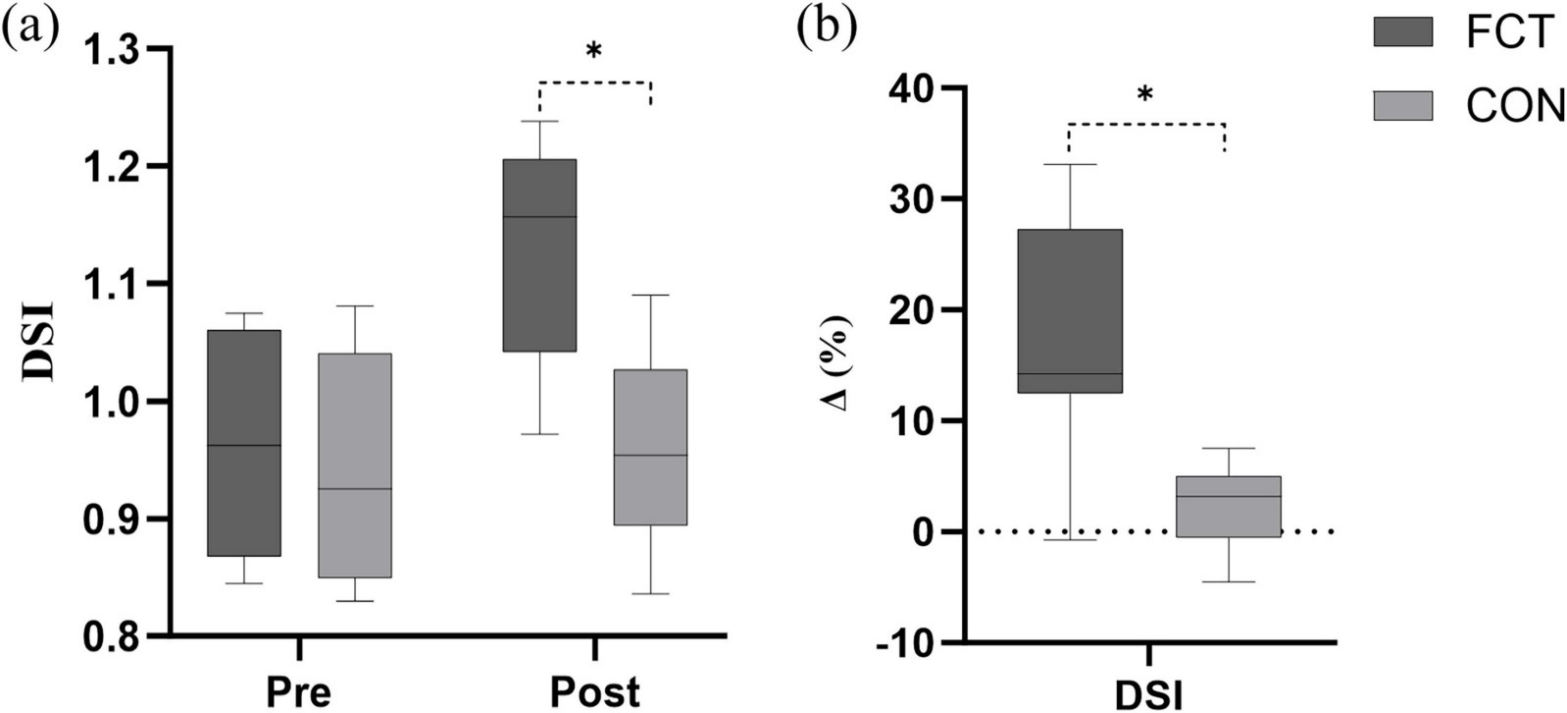
Effect of FCT on the DSI and the DSI growth rate. **(a)** DSI, **(b)** growth rate.

A significant interaction effect between time and group was observed (F = 29.47, *p* < 0.001). The adjusted difference in change was 0.147 (*p* < 0.001, 95% CI: 0.092–0.215). The main effect of time showed a significant difference (F = 46.92, *p* < 0.001). The main effect of the group showed a significant difference (F = 36.77, *p* < 0.001) (Figure 10a; Table 2).

An independent *t*-test comparing the Δ% in DSI revealed a significant difference between the groups (*t* = 5.06, *p* < 0.001, 95% CI: 9.21–21.98, *d* = 2.07, large effect) (Figure 10b).

## 4 Discussion

This study presents the first evidence that FCT enhances lower limb strength and power in elite martial arts athletes. These findings showed that, compared to traditional resistance training, FCT significantly improved MFO, relative MFO, and RFD during IMTP and improved CMJ and SJ height, PPO, MPO, and DSI, suggesting that FCT could be an effective training strategy for elite martial arts athletes.

The improvements in IMTP performance could be attributed to enhanced neural drive and motor unit recruitment (29). By combining heavy compound exercise with plyometric and ballistic exercises within a single session, FCT stimulates adaptations across the full of the F–V curve and elicits PAPE (30), transient potentiation of force output mediated by increased α-motoneuron excitability, heightened cortical activity, and phosphorylation of myosin regulatory light chains (31). Repeated exposure to this neural priming stimulus can lead to chronic adaptations, manifesting as increased maximal isometric strength and RFD (32). Cormier et al. demonstrated in their meta-analysis that the acute enhancement induced by PAPE can lead to significant chronic improvements in maximal strength and RFD (33). Previous studies primarily focused on how FCT improves the peripheral mechanism (13–17, 19, 21, 24, 34, 35); future studies should employ surface electromyography or transcranial magnetic stimulation to quantify changes in neural drive following FCT interventions.

The improvements in CMJ and SJ performance are consistent with previous studies. For instance, Rebelo et al. demonstrated that a 6-week FCT intervention can enhance the reactive strength index and CMJ performance in young athletes (19). Hernández-Preciado et al. found acute vertical jump potentiation after a single FCT session in recreational populations (34). The improvements in CMJ and SJ performance are likely due to the FCT protocol, which provides comprehensive neuromuscular stimulation across the full spectrum of the F–V curve (36). Specifically, heavy compound exercises increase maximal force output by enhancing neural drive and recruiting high-threshold motor units, thereby shifting the curve upward in the high-force, low-velocity domain (37). Plyometric and light-to-moderate load compound exercises target the moderate and high-velocity domains, facilitating improvements in RFD, motor unit firing rates, and muscle–tendon stiffness (38, 39). These combined effects likely result in enhanced excitation–contraction coupling, improved synchronization of agonist muscle activity, and more efficient utilization of both contractile and elastic elements during dynamic lower limb actions (40–43). Furthermore, repeated high-velocity exercises may induce morphological adaptations such as increased muscle fiber pennation angle (44), enhanced tendon compliance (45), and optimized muscle–tendon interaction, all of which are critical for power expression during explosive tasks (40).

Interestingly, although both CMJ and SJ performance improved, the relative gains were more pronounced in the SJ (PPO Δ 6.04%, MPO Δ 7.89%) than in the CMJ (PPO Δ 2.00%, MPO Δ 2.64%). This divergence likely reflects the distinct biomechanical and neuromuscular demands of the two jump types (26). The SJ begins from a static position and isolates concentric muscle action, requiring high levels of force generation without any contribution from elastic recoil or stretch reflexes (46). Thus, its improvement is closely linked to enhancements in intrinsic muscle contractile properties, neural drive (47), and intramuscular coordination (48), which are powerfully elicited by the heavy and ballistic loading components of FCT (49). In contrast, the CMJ incorporates a rapid eccentric phase preceding the concentric action, engaging the SSC and relying more on tendon stiffness, muscle–tendon synergy, and spinal reflex excitability (50, 51). The assisted plyometric exercises in FCT may have enhanced SSC efficiency by improving storage recoil behavior and reflex potentiation (52). This may be due to a ceiling effect in these elite athletes who already have efficient SSC utilization. The ceiling effect, defined as the reduced potential for measurable improvements in athletes who already perform at or near their physiological and biomechanical limits, can significantly attenuate observable gains from additional training interventions (53). Alternatively, the short inter-exercise rest intervals in FCT (30–40 s) may have resulted in residual neuromuscular fatigue during the plyometric phases, slightly dampening the acute potentiation of SSC-related performance (54, 55). Furthermore, the neural control strategies differ between the SJ and the CMJ: the SJ requires precise motor unit recruitment and high discharge rates to initiate movement from a static state, whereas the CMJ involves complex coordination of braking and propulsive phases (26). The DSI result complements the larger improvements observed in the SJ than in the CMJ, supporting the interpretation that FCT primarily strengthened concentric, task-specific force production. These differences suggest that FCT-induced PAPE may preferentially benefit concentric performance, as reflected in the larger improvements in the SJ metrics. This is consistent with previous studies. For instance, Sun et al. reported that the SJ exhibited greater improvements than the CMJ under PAPE conditions (56). In summary, these findings suggest that FCT induces a more substantial training stimulus on concentric dominant actions. FCT is an effective method for converting maximal strength into dynamic jump performance. To further optimize SSC efficiency, training should focus on reactive plyometrics, rapid stretch loading, and eccentric control, thereby challenging the pre-stretch phases. This has important implications for sport-specific performance, particularly in martial arts, where athletes must execute forceful movements from static or transitional postures with minimal preparatory loading. Practically, coaches can use FCT to enhance lower limb strength and power, scheduling two weekly sessions with at least 48 h of recovery. Future studies should further dissect these mechanisms using time–force curve analysis, ultrasound-based muscle–tendon assessment, and reflex latency testing to clarify how different FCT components contribute to concentric vs. SSC-driven performance gains.

### 4.1 Limitations

This study has several limitations. It involved a small sample of elite male martial artists, limiting its generalizability and neglecting gender differences. The observed effects were often small, necessitating cautious interpretation. In addition, the absence of physiological and neuromuscular biomarkers constrained mechanistic understanding, and the lack of fatigue response data hindered a comprehensive view of training adaptations. Future research should include larger, more diverse samples, explore gender-specific responses, incorporate relevant biomarkers, and employ longitudinal designs to assess the effect of FCT on combat athletes.

## 5 Conclusion

This study provides preliminary evidence that FCT may have advantages over traditional resistance training in improving lower limb strength and power among elite martial arts athletes. These findings imply a possible role for FCT within periodized training programs. However, due to the small sample size, the exclusive focus on elite male athletes, and the limited magnitude of some effects, the results should be interpreted with caution. Future research should confirm these findings across different martial arts disciplines, genders, and training levels, and examine the long-term effects of FCT using biomechanical and physiological measures.

## Data Availability

The original contributions presented in the study are included in the article/**Supplementary Material**, further inquiries can be directed to the corresponding authors.
