# Supplementary material for: The effects of an 8-week French Contrast Training program on lower limb strength and power in elite martial arts athletes
Source: Front Sports Act Living. 2025 Oct 24;7:1686891. doi: 10.3389/fspor.2025.1686891 (PMC12592053; doi:10.3389/fspor.2025.1686891)
Supplement: Supplementary file 1 [file Datasheet1.pdf]

Supplementary Material

The Effects of an Eight-Week French Contrast Training Program on Lower-Limb Strength and Power in Elite Martial Arts Athletes

Hao Chen[1, †], Ziren Zhao[2, †], Xin Zheng[2], Liquan Cao[3], Jingtao Du[4, *], Zhongtao Yu[5, *]

[1] Tianjin Vocational College of Sports, Tianjin, China;

[2] College of Physical Education and Health Science, Chongqing Normal University, Chongqing, China;

[3] Tianjin University of Sport, Tianjin, China;

[4] College of Sports and Health, Chengdu University of Traditional Chinese Medicine, Chengdu, Sichuan, China;

[5] Tianjin Institute of Sports Science, Tianjin Sports Comprehensive Guarantee Center, Tianjin, China.

*** Correspondence:**
Zhongtao Yu, Tianjin Institute of Sports Science, Tianjin Sports Comprehensive Guarantee Center, No.17 Tu'an New Town, Jinghai District, Tianjin, China; e-mail address: tjgmtzjc@163.com. Jingtao Du, College of Sports and Health, Chengdu University of Traditional Chinese Medicine, No. 1166, Liutai Avenue, Wenjiang District, Chengdu, Sichuan, China; e-mail address: 13880295720@163.com.

† **Equal contribution and first authorship:** These authors contributed equally to this work and share first authorship

1 Supplementary Tables

1.1 Table S1 The outcomes of pre-experimental.

1.2 Table S2 Warm-up strategy.

1.1 Table S1 The outcomes of pre-experimental.

Variable	Group	Pre	Post	Δ% (Mean)	Cohen's d	*P*	F
	**MFO (N)**						
	FCT	2368.04±714.48	2634.50±891.22	10.47	0.504	*0.059*	4.682
	CON	2257.55±578.89	2261.63±549.10	0.51			
	**RMFO (N/kg)**						
**IMTP**	FCT	3.94±0.66	4.40±0.60	12.13	0.958	*0.058*	4.735
	CON	3.69±0.37	3.85±0.55	3.87			
	**RFD (N/s)**						
	FCT	3432.50±1055.50	3869.30±913.10	13.08	0.665	*0.003*	15.652
	CON	3241.12±970.29	3263.04±910.17	0.93			
	**Height (cm)**						
	FCT	48.28±8.49	50.41±8.51	4.57	0.203	*0.002*	17.801
	CON	46.48±11.52	47.08±11.29	1.47			
	**PPO (W)**						
**CMJ**	FCT	4219.83±1214.28	4293.67±1183.91	2.04	-0.284	*0.030*	6.630
	CON	4653.67±1381.31	4655.67±1359.76	0.22			
	**MPO (W)**						
	FCT	2298.33±573.04	2361.17±584.57	2.78	-0.082	*0.046*	5.360
	CON	2423.67±595.86	2408.17±564.75	-0.31			
	**Height (cm)**						
	FCT	53.80±7.03	55.45±6.24	3.26	0.681	*0.007*	12.082
	CON	49.68±12.10	49.00±11.85	-1.31			
	**PPO (W)**						
**SJ**	FCT	5337.00±702.08	5537.00±702.08	3.55	0.504	*0.079*	3.910
	CON	4984.50±1313.89	4977.50±1320.95	-0.19			
	**MPO (W)**						
	FCT	2723.67±632.22	2862.33±653.27	5.26	0.496	*0.014*	9.147
	CON	2477.00±891.18	2472.67±899.84	-0.23			
**EI**	FCT	-2.65±18.12	-2.09±17.02	0.56	-0.443	*0.235*	1.616
	CON	5.65±27.98	8.92±30.79	3.27			
**DSI**	FCT	0.96±0.09	1.15±0.07	20.00	2.569	*<0.001*	36.990
	CON	0.94±0.10	0.97±0.07	2.84			

CMJ, countermovement jump; **DSI**, dynamic strength index; **EI**, elasticity index; **IMTP**, Isometric Mid-Thigh Pull; **MFO**, maximal force output; **MPO**, mean power output; **PPO**, peak power output; **RMFO,** relative maximal force output; **SJ**, squat jump

1.2 Table S2 Warm-up strategy

Content	Dose
Jog, lateral shuffle	3 minutes
Ankle rocker, back squat, hip flex flow	2 sets with 6-8 repetitions
Side plank, mini-band lateral walk	2 sets with 6-8repetitions
30 cm drop jump	2 sets with 6-8repetitions
